# The hoverflies of the Dauzet collection at the Musée des Confluences in Lyon (Diptera, Syrphidae)

**DOI:** 10.3897/BDJ.13.e146082

**Published:** 2025-03-28

**Authors:** Gabriel Nève, Jocelyn Claude, Harold Labrique

**Affiliations:** 1 Aix Marseille University, Marseille, France Aix Marseille University Marseille France; 2 IMBE, Marseille, France IMBE Marseille France; 3 CNRS, Marseille, France CNRS Marseille France; 4 Avignon University, Avignon, France Avignon University Avignon France; 5 IRD, Marseille, France IRD Marseille France; 6 Independent researcher, Chissey-lès-Mâcon, France Independent researcher Chissey-lès-Mâcon France; 7 Musée des Confluences, Lyon, France Musée des Confluences Lyon France

**Keywords:** Diptera, Syrphidae, distribution, collection, France

## Abstract

**Background:**

Maurice Dauzet collected hoverflies mainly in Loire and Haute-Loire from 1980 to 2017. Here, we provide the data from his hoverfly collection and card records, in order to support efforts towards a better understanding of the changing distribution of pollinators.

**New information:**

The hoverflies from the Dauzet collection includes 1302 specimens and 423 additional records written on cards, totalling 1725 data of 221 species. Data from these specimens and records are presented here, with date of capture and location details. The collection contains specimens of species endangered at the European level: *Cheilosiagagatea*, *Epistropheleiophthalma*, *Paragusalbifrons* and *Paragusfinitimus*. The Dauzet collection adds 81 species data to the known departmental distribution of the hoverflies of France. This revision invalidates data on eight species in the Loire Department previously published by Maurice Dauzet.

## Introduction

Pollinator decline has been documented worldwide (e.g. Raven and Wagner (2021)), including hoverflies (Hallmann et al. 2021). Amongst Diptera, the hoverflies (Syrphidae family) are the most important pollinators (Gilbert 1980, Rotheray and Gilbert 2011, Dunn et al. 2020), although other families are important as well, albeit often neglected (Orford et al. 2015). Efficient conservation relies on the knowledge of past and present distribution of the species involved. We wish to present here all the hoverfly records Maurice Dauzet gathered during his lifetime, including both his specimens and his card records, with full data.

### Maurice Dauzet short biography

**Figure 1.**
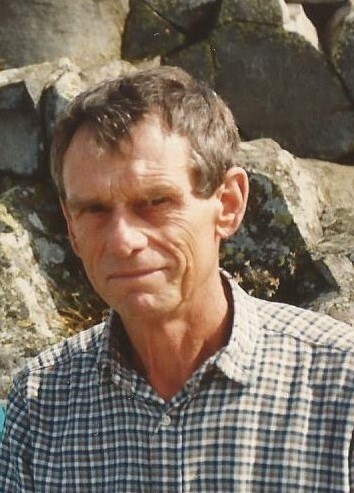
Portrait of Maurice Dauzet (photograph supplied by Isabelle Cuzor).

Maurice Dauzet (Fig. 1) was born on 14 December 1927. After a career as an engineer in the industrial sector, he took early retirement in the 1980s. One of his sons sent him some insects from French Guyana, which greatly interested him. He then set about studying the insects of his region, in particular Hymenoptera and Diptera and, secondarily, Coleoptera, Heteroptera and Homoptera. He lived in Saint-Etienne (Loire) and had joined the Société de Sciences Naturelles Loire Forez and the Société linnéenne de Lyon. Within the former, he took part in the Loire Biodiversity Inventory (Dauzet et al. 2015)

**Figure 2.**
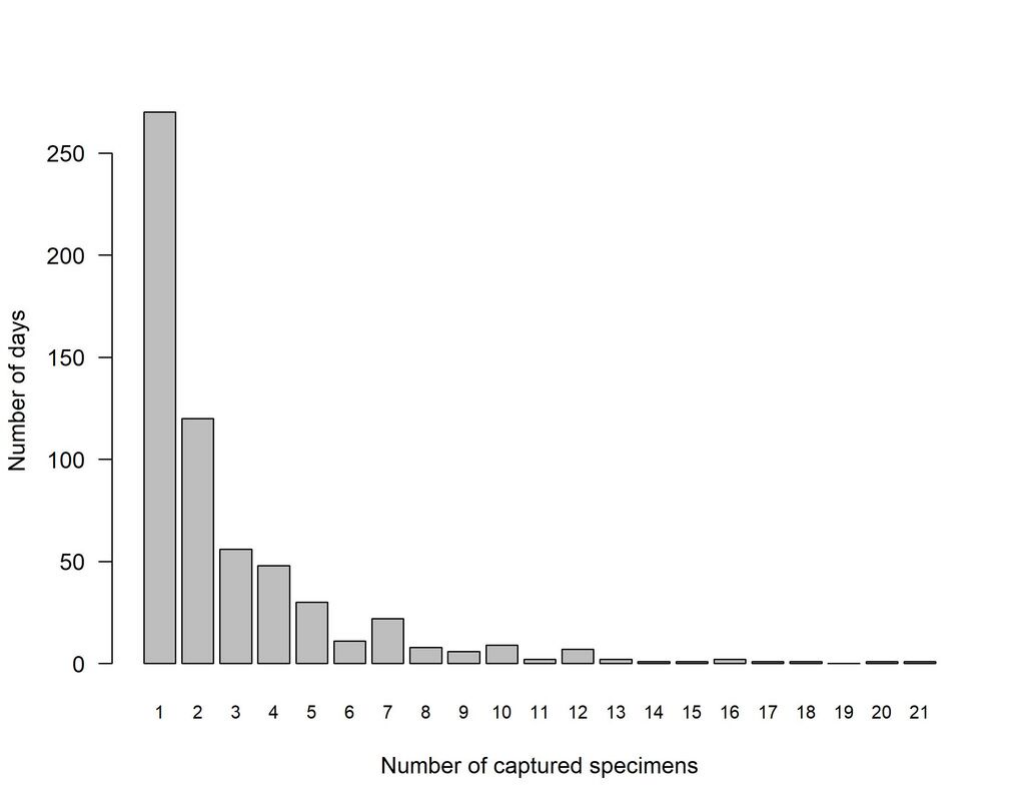
Number of capture days per number of individuals caught on a single day.

His first hoverfly specimen dates back to 1980 and his collection of hoverflies began in 1986, with captures up to 2017 when he was 89 years old! He collected on all days of the week and retained only what interested him: on 270 of the 599 days on which at least one hoverfly specimen was captured, only one specimen was recorded and, on 28 days, more than ten specimens were retained, up to 21 specimens (Fig. 2). Whenever possible, he tried to secure a pair of each species caught in the same place on a given day, which happened 125 times. He captured nearly all the specimens present in his collection; his friend Christan Bellut gave him 28 specimens, one specimen come from André Ulmer and one from an unidentified person named Jallieu. Dauzet's identifications proved correct in most cases. He worked mainly alone on his collection, gathering the necessary paper reprints by post from the authors. He did not use a computer, but used a card system to keep a record of the specimens he had caught. These cards, now in the Musée des Confluences, Lyon, were used by Dauzet to keep track of all his hoverfly records, whether he kept the specimens or not, using a code system to indicate whether each specimen was retained or not. One or more cards were used per species. For each species, the referenced used for identification are mentioned; these are usually Verrall (1901), Sack (1935), Séguy (1961) and van Veen (2004), supplemented for a few species by Speight and Sarthou (2011) and Speight (2013), for *Pipiza* and *Xanthogramma* genera and by Goeldlin de Tiefenau et al. (1990) for *Platycheirus*. His friends Patrick Subit and Justin Galtier helped him to organise his data and to publish a summary of the distribution data he had gathered in the Loire Department (Dauzet et al. 2015).

Many of his specimens come from Saint-Pierre-Eynac (Haute-Loire), where his family had a second home. The labels of specimens from this locality bear red triangles on two corners and usually only an abbreviation of the locality name. He had the habit of always writing the code number of the Department of capture on the labels, which made it possible to find the localities without ambiguity.

His specimen preparations were very meticulous and he usually prepared the genitalia of males, even of common and otherwise easy-to-identify species such as *Eristalistenax*. This proved very useful for identifying *Pipizella* and *Paragus*, where criteria, based on genitalia, are paramount for reliable identification (Goeldlin de Tiefenau 1976, van Steenis and Lucas 2011).

On his retirement into a nursing home, in 2019, Dauzet donated his insect collection and his written insect records on cards to the Musée des Confluences, where they remained to this day. Only the Coleoptera collection remained in his family. He was aware of the research we carried out on his bee collection at the Musée des Confluences (Meunier et al. 2023), even though his health no longer allowed him to take part. He died on 26 December 2021, leaving four children, seven grandchildren and four great-grandchildren. His scientific documentation was donated to the Société de Sciences Naturelles Loire Forez and his personal entomological archives to the Musée des Confluences (Lyon), along with his entomological collection. His insect collection has become a testimony to the biodiversity of insects in the Loire Department and surrounding area at the beginning of the 21^st^ century.

## General description

### Purpose

The dataset we publish here includes data on 1725 hoverfly specimens collected between 1980 and 2017. All were identifed at least to genus level and most often to species level. The aim of the present paper is to publish this dataset, of which only a small part Dauzet presented in his synthesis of the data of the Loire Department (Dauzet et al. 2015).

## Sampling methods

### Sampling description

For each specimen, the data written on the label were retrieved: location and date of capture and sometimes the plant on which the specimen was caught. All specimens were assigned a unique identification code which was written on a label added below Dauzet's labels and the new identication label. Additional data retreived from the handwritten card were input with location and dates as written by Dauzet.

### Quality control

All specimens were identified by GN or JC, mainly from the recent general hoverfly identification works (van Veen 2004, Speight and Sarthou 2017, Bot and Van de Meutter 2023) and more specialised references (Goeldlin de Tiefenau 1976, van der Goot 1981, Barkalov and Ståhls 1997, Speight and Langlois 2020, Speight et al. 2021). We also compared Dauzet's specimens with specimens of our reference collections.

Nomenclature follows the recent Atlas of the Hoverflies of France (Speight et al. 2024) and the IUCN Red List of European Syrphidae (Vujić et al. 2022), even if some name changes have been already adopted by Bot and Van de Meutter (2023), notably for *Cheilosiasoror*, *Chrysotoxumfasciatum* and *Parasyrphusvittiger* which they name *Cheilosiaruffipes*, *Chrysotoxumarcuatum* and *Parasyrphusrelictus*, respectively.

### Step description

GN and JC re-identified all specimens in the collection and added their own identification labels, as well as a label with an individual Museum code. We ended up changing the name given to 184 of his 1302 specimens. The most common reason for name changes were due to the downgrade to genus level of specimens identified to species level by Dauzet (69 cases). Dauzet wrote *Cheilosiaalbitarsis* on the labels of females in the *Cheilosiaalbitarsis*/*C.ranunculi* complex, although he mentionned on the card for this species that females of this species pair are unidentifiable (Doczkal 2000). Dauzet also assigned specimens to individual species in the *Microdonmyrmicae*/*M.mutabilis* complex. We refrained from giving species names to most females in the genera *Paragus*, *Pipizella*, *Pipiza*, *Merodon*, *Eumerus*, *Platycheirus* and *Sphaerophoria*. The one specimen from French Guyana could be assigned to the genus *Copestylum* thanks to Reemer (2016).

The update of nomenclature was the second most common reason of name change (60 cases: e.g. *Meligrammaeuchroma* changed into *Epistrophellaeuchroma* and *Eristalisinterrupta* changed into *Eristalisnemorum*). We had to correct the identification given by Dauzet in 47 cases. A few specimens not identified to species level by Dauzet could be positively assigned to a species (7 cases) or to a genus (1 case).

If only a tentative identification were given, 'cf.' is indicated in the 'identificationRemarks' column and a value of 0 was given in the 'identificationVerificationStatus' column. All data backed only by Dauzet's handwritten cards were also given an identificationVerificationStatus of 0. All French localities were located using the geoportail.gouv.fr website from which the longitude and latitude were retrieved. Coordinates of other localities were found using Google Earth Pro. Whenever Dauzet used place names, the coordinate uncertainty was input at 1000 m. If only the locality were known, then a value of 5000 m was input. All formats follow GBIF Darwin Core specification, to ensure interoperability with other international databases.

## Geographic coverage

### Description

**Figure 3.**
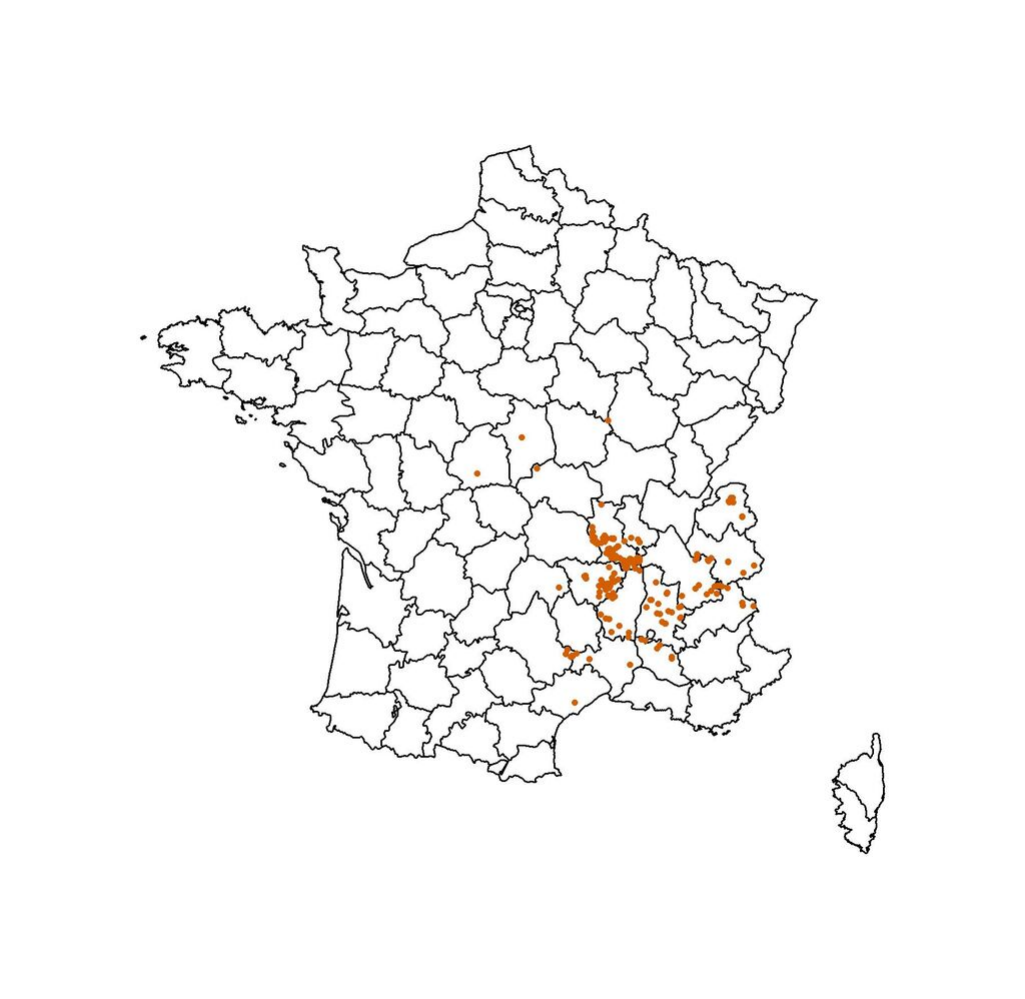
Distribution of hoverfly specimens captured by Maurice Dauzet. One specimen from French Guyana, one from Italy and one from Greece are omitted.

Most of Dauzet's specimens come from France (Fig. 3). The only exceptions are one specimen from French Guyana (5.4232°N, 54.0851°E), one from Italy (Susa, 45.1431°N, 7.0611°E) and one from Greece (35.6100°N, 23.5782°E). In France (coordinate range below), most of the records come from the Loire (843 specimens) and Haute-Loire (247 specimens) Departments (Table 1).

### Coordinates

43.4366 and 47.3196 Latitude; 1.5091 and 6.9931 Longitude.

## Taxonomic coverage

### Description

The dataset describes the data of 1725 specimens belonging to 221 Syrphidae species (Table 2)

### Taxa included

**Table:** 

Rank	Scientific Name	Common Name
kingdom	Animalia	Animals
subkingdom	Eumetazoa	
phylum	Arthropoda	Arthropods
subphylum	Pancrustacea	
class	Insecta	Insects
subclass	Pterygota	
order	Diptera	Flies
suborder	Brachycera	
superfamily	Syrphoidea	
family	Syrphidae	Hoverflies

## Temporal coverage

**Data range:** 1986-5-17 – 2018-4-26.

### Notes

**Figure 4.**
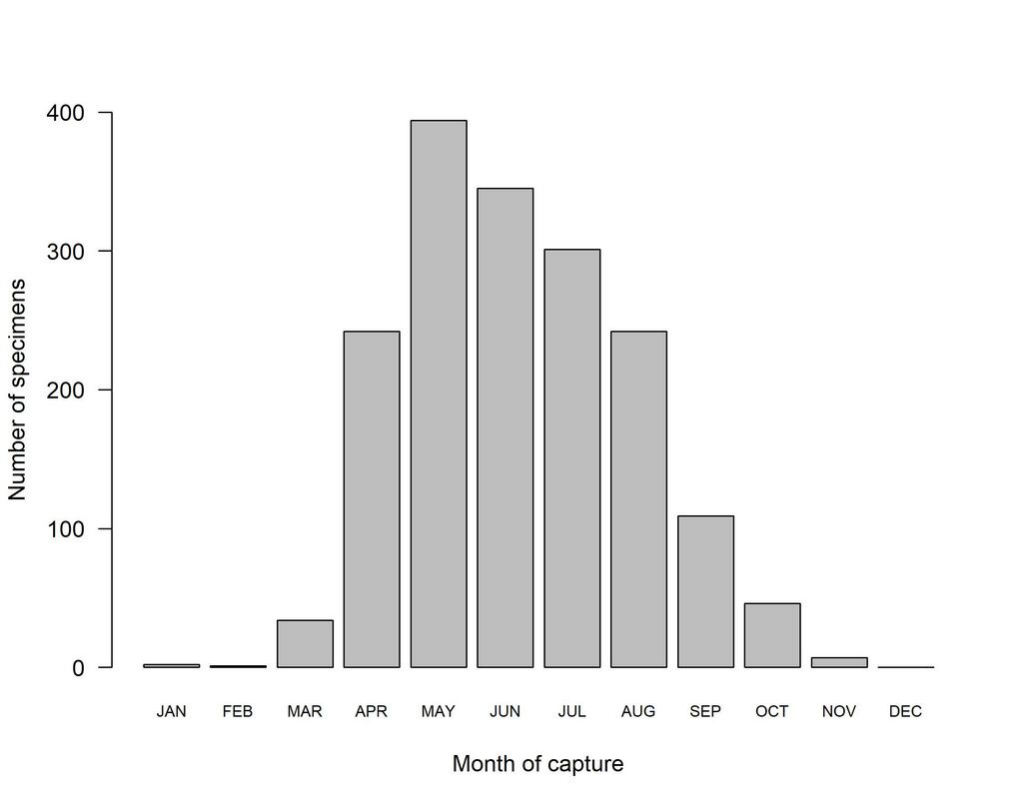
Number of hoverfly specimens captured per month by Maurice Dauzet.

**Figure 5.**
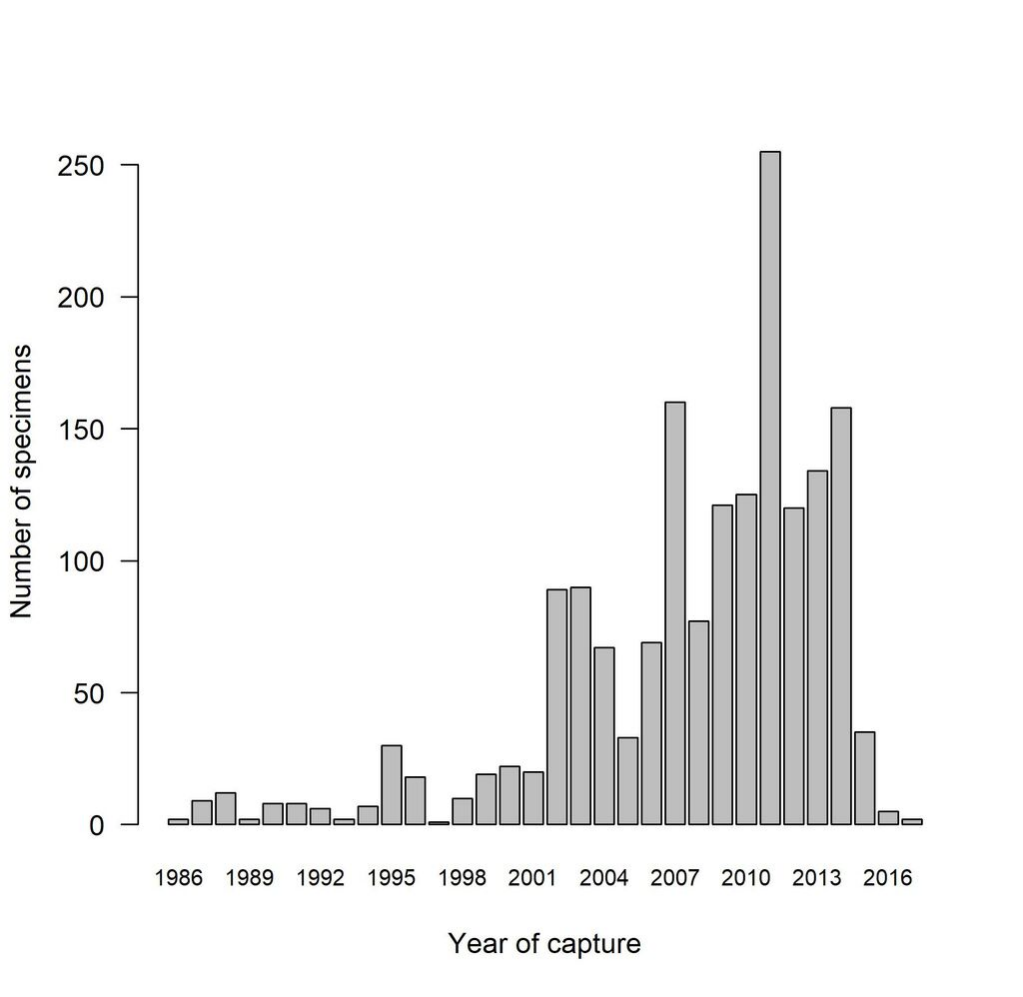
Numbers of hoverfly specimens captured per year by Maurice Dauzet. One specimen from 1980 is omitted.

One additional specimen from 1980 only bears year collection information. Specimens were collected all months of the year, with the highest number of specimens caught from May to July (Fig. 4). Dauzet was most active collecting hoverflies from 2002 to 2014, from the age of 74 to the age of 86 years old (Fig. 5)!

## Collection data

### Collection name

Maurice Dauzet insect collection

### Collection identifier

Dauzet

### Parent collection identifier

Insects

### Specimen preservation method

Dried and pinned specimens

### Curatorial unit

Centre Louis Lortet, Musée des Confluences, Lyon. Contact: Harold Labrique (email: harold.labrique[at]museedesconfluences.fr)

## Usage licence

### Usage licence

Creative Commons Public Domain Waiver (CC-Zero)

### IP rights notes

This work is licensed under a Creative Commons Attribution (CC-BY) 4.0 Licence. All work derived from the present study should cite it appropriately, including the Museum where the material is held.

## Data resources

### Data package title

Syrphidae Dauzet collection

### Resource link

https://doi.org/10.5281/zenodo.14602286

### Number of data sets

1

### Data set 1.

#### Data set name

Maurice Dauzet Syrphidae collection

#### Data format

CSV (tab delimited values)

#### Character set

Syrphidae_Dauzet_v02.csv

#### Download URL

https://doi.org/10.5281/zenodo.14602286

#### Data format version

Darwin core, so that it may be transferred later into GBIF.

#### Description

The dataset includes data on 1725 hoverfly individuals collected by Maurice Dauzet, in GBIF compatible format; 1302 of these specimens are hosted in his collection and 423 come from his record data kept on handwritten cards.

**Data set 1.** 

Column label	Column description
occurrenceID	Individual identification code. Each specimen bears a label with this code. Records from his handwritten cards have a code including the word "fiches" (French word for cards).
basisOfRecord	The specific nature of the data record (i.e. PreservedSpecimen in the case of pinned specimen or MaterialEntity in the case of written record only, as presented later in this table).
eventDate	Event date in the format YYYY-MM-DD if the capture date is known to the date or YYYY-MM if only the month and year are known or YYYY if only the year is known. If the specimen was captured during a time lapse in which the first and last days are known, then these two dates are given separated by a slash bar (/), e.g. 2015-06-02/2015-07-15.
year	Year of capture.
month	Month of capture.
day	Day of capture.
verbatimEventDate	Date of capture, as mentioned on the label or on the card.
kingdom	Kingdom name (i.e. Animalia).
phylum	Phylum name (i.e. Arthropoda).
class	Class name (i.e. Insecta).
order	Ordername (i.e. Diptera).
family	Family name (Syrphidae).
previousIdentifications	Species name originally given on the specimen label by Maurice Dauzet.
genus	Genus name.
specificEpithet	Species epithet of the scientificName.
scientificNameAuthorship	Name of the scientist who first described the species and year of publication of the description.
scientificName	Lowest taxonomic rank possible given to the specimen, usually the species name, sometimes the genus, with author and year.
sex	Male (M) or female (F).
taxonRank	Species or genus.
identificationQualifier	In case the identification could be given only to a species group, 'cf.' was input.
identificationRemarks	Any comment on the identification of the specimen, with list of possible species. Comments by Dauzet on the label are indicated with his initials (MD).
IdentifiedBy	Name of the entomologist who identified the specimen.
dateIdentified	Year of most recent identification.
identificationVerificationStatus	Whether (coded 1) or not (coded 0), the identification could be checked on a specimen. Data from cards, from Dauzet's own identifications only, were coded 0. Specimens assigned to species group were also coded 0.
country	Country of capture.
countryCode	Two letter country code of the specimen capture location.
stateProvince	French Departmental administrative division.
locality	Location of capture, usually the municipality.
verbatimLocality	Any geographical indication, as it is written on the label or card.
decimalLatitude	Geographic latitude (in decimal degrees) of the capture location.
decimalLongitude	Geographic longitude (in decimal degrees) of the capture location.
coordinateUncertaintyInMetres	Uncertainty in coordinates, in metres.
recordedBy	Name of collector (i.e. legitimate information; i.e. usually Dauzet, Maurice)
occurrenceRemarks	Any ecological data or comment on the label.
minimumElevationInMetres	Lower limit of the range of altitudes indicated on the label.
maximumElevationInMetres	Higher limit of the range of altitudes indicated on the label.
geodeticDatum	System and set of reference points upon which the geographic coordinates are based (i.e. WGS84).
georeferencedBy	Identity of the person who added the latitude and longitude data, i.e. Nève, Gabriel.
georeferenceProtocol	How the georeference was computed, i.e. from label data (verbatimLocality).
georeferenceSources	Georeference code was inferred from geoportail.gouv.fr.
georeferencedDate	Georeference work was performed in 2024.
institutionCode	Institution where the specimen is held (i.e. MDC: Musée des confluences, previously known as MHNL: Muséum d'Histoire Naturelle de Lyon).
CollectionCode	Collection code within the Museum, i.e. M. Dauzet.
catalogNumber	Combination of box number and individual specimen number.
organismQuantity	Number of individuals bearing the same label (i.e. 1).
organismQuantityType	Individuals.
language	The dataset is mainly written in French, apart from column headings, which are in English.
associatedReferences	Any reference citing the relevant specimen.
materialEntityID	For records found in Dauzet's written records only, these referring to his handwritten cards.

## Additional information

Publication of data is paramount to back up conservation efforts for biodiversity preservation. Collectors end up publishing some of their data in their lifetime (Nève et al. 2024). The present publication is part of large programme towards a better knowledge of the French hoverfly past and present distribution (Speight et al. 2024). We add here 81 Departmental distribution records (Table 3), mainly from the Haute-Loire Department, which was previously poorly known.

The three specimens of *Cheilosiacaerulescens* were captured near the country home of the Dauzet family, at Saint-Pierre-Eynac, Lardeyrol (Loire), at an altitude of ca. 835 m, a figure low for this mountain species, which depends on mountain *Sempervivum* sp. (Speight 2020). The question remains open as to the recent records at low altitude coming from a natural colonisation of these habitats by this alpine species or whether low altitude specimens come from specimens imported with transplanted *Sempervivum* sp., as in the case of British records, which most probably originate from an introduction (Collins and Halstead 2008) and are now widespread in British suburban gardens (Ball and Morris 2024).

The data gathered from Dauzet's specimens and handwritten cards were compared with the published records. Some of the dates mentionned in Dauzet et al. (2015) were corrected, such as a *Cheilosiaalbipila* specimen mentioned from March, which was captured in May, according to the label data.

Records of eight species mentioned from the Loire Department by Dauzet et al. (2015) have to be corrected. Four species mentioned from the Loire Department were, upon examination, based on misidentified material: *Cheilosiabracusi*, *Neoasciaannexa*, *Riponnensiasplendens* and *Melangynabarbifrons*. The specimens of *Melangynacompositarum* and *Paragusflammeus* could not be located in the collection. The specimens originally identified as *Sphaerophoriaestebani* and *S.philanthus* by Dauzet could not by confirmed as such, as they were either females (3 cases) or damaged (1 male *S.philanthus*). If the presence of these species in the Loire Department, as depicted by Speight et al. (2024), is not backed by any material other than Dauzet's, these Departmental data should be deleted. Speight et al. (2024) did not take into account Dauzet's Loire Department records of *Cheilosiabracusi* and *Paragusflammeus*, presumably because these were atypical records of species notoriously difficult to identify.

The only record of *Chrysotoxumcisalpinum*, from Montmaur-en-Diois, Drôme Department, on 12 Aug 1996, was based on a specimen shown to him, but not retained in his collection. The card record mentions that the identication of this specimen was based on Sack (1935), p. 224, on Séguy (1961), p. 118 and on van Veen (2004), p. 81.

Some of the species mentionned here are rare. *Epistropheobscuripes* is known in France only from two Departments (Speight et al. 2024): Loire and Haute-Saône, with Loire data being the previously-published Dauzet specimen (Dauzet et al. 2015).

Amongst the species captured by Dauzet, five are considered endangered in Europe: *Cheilosiagagatea*, *Cheilosiapictipennis*, *Epistropheleiophthalma*, *Paragusalbifrons* and *Paragusfinitimus*. Three are considered vulnerable: *Chalcosyrphusfemoratus*, *Chrysotoxumcisalpunum* and *Microdonmutabilis*. Seven are near threatened: *Chrysogastervirescens*, *Chrysotoxumelegans*, *Chrysotoxumoctomaculatum*, *Eupeodestirolensis*, *Merodonflavus*, *Microdonanalis* and *Pyrophaenagranditarsa*.

It is hoped that publishing data on these species will help defining their distribution and, hence, define effective conservation measures.

## Figures and Tables

**Table 1.** Numbers of hoverfly specimens recorded by Maurice Dauzet per French Department, including both specimens retained in is collection and records written on cards.

French department	Number of records
Allier	2
Ardèche	29
Aveyron	6
Cantal	2
Cher	1
Drôme	60
Gard	6
Guyane	1
Haute-Loire	335
Haute-Savoie	26
Hautes-Alpes	12
Hérault	2
Indre	1
Isère	51
Loire	1118
Nièvre	1
Rhône	21
Savoie	23
Vaucluse	25
[unknown]	1

**Table 2.** Hoverfly recorded by Maurice Dauzet. "*S*pecimens" refer to specimens in his collection and "card records" refer to records present only on his written cards. IUCN status according to Vujić et al. (2022): LC: Low Concern, EN: Endangered, VU: Vulnerable, NT: Near Threatened.

**Species or genus**	**European IUCN status**	**Number of specimens**	**Card records**	
*Bacchaelongata* (Fabricius, 1775)	LC	1	0	
*Brachyopapanzeri* Goffe, 1945	LC	1	0	
*Brachyopatestacea* (Fallén, 1817)	LC	3	0	
*Brachypalpoideslentus* (Meigen, 1822)	LC	1	0	
*Brachypalpuslaphriformis* (Fallén, 1816)	LC	1	0	
*Brachypalpusvalgus* (Panzer, 1798)	LC	4	2	
*Chalcosyrphusfemoratus* (Linnaeus, 1758)	VU	1	0	
*Chalcosyrphusnemorum* (Fabricius, 1805)	LC	2	0	
*Chalcosyrphusvalgus* (Gmelin, 1790)	LC	1	0	
*Cheilosia* Meigen, 1822		6	0	
*Cheilosiaaerea* Dufour, 1848	LC	6	0	
*Cheilosiaalbipila* Meigen, 1838	LC	9	0	
*Cheilosiaalbitarsis* (Meigen, 1822)	LC	34	4	
*Cheilosiaantiqua* (Meigen, 1822)	LC	4	0	
*Cheilosiabarbata* Loew, 1857	LC	24	0	
*Cheilosiabergenstammi* Becker, 1894	LC	1	0	
*Cheilosiacaerulescens* (Meigen, 1822)	LC	3	0	
*Cheilosiacanicularis* (Panzer, 1801)	LC	6	0	
*Cheilosiacynocephala* Loew, 1840	LC	3	0	
*Cheilosiaderasa* Loew, 1857	LC	3	0	
*Cheilosiaflavipes* (Panzer, 1798)	LC	2	0	
*Cheilosiafraterna* (Meigen, 1830)	LC	8	0	
*Cheilosiagagatea* Loew, 1857	EN	1	0	
*Cheilosiagigantea* (Zetterstedt, 1838)	LC	2	0	
*Cheilosiahimantopus* (Panzer, 1798)	LC	3	0	
*Cheilosiaillustrata* (Harris, 1780)	LC	12	9	
*Cheilosiaimpressa* Loew, 1840	LC	2	0	
*Cheilosialaticornis* Rondani, 1857	LC	3	0	
*Cheilosialatifrons* (Zetterstedt, 1843)	LC	3	0	
*Cheilosialenis* Becker, 1894	LC	18	0	
*Cheilosiamelanura* Becker, 1894	LC	1	0	
*Cheilosiamutabilis* (Fallén, 1817))	LC	3	0	
*Cheilosianigripes* (Meigen, 1822)	LC	2	0	
*Cheilosiaorthotricha* Vujic & Claussen, 1994	LC	1	0	
*Cheilosiapagana* (Meigen, 1822)	LC	7	1	
*Cheilosiapersonata* Loew, 1857	LC	7	0	
*Cheilosiapictipennis* Egger, 1860	EN	1	0	
*Cheilosiaproxima* (Zetterstedt, 1843)	LC	3	0	
*Cheilosiaranunculi* Doczkal, 2000	LC	5	0	
*Cheilosiarhynchops* Egger, 1860	LC	1	0	
*Cheilosiasahlbergi* Becker, 1894	DD	1	0	
*Cheilosiascutellata* (Fallén, 1817)	LC	1	0	
*Cheilosiasoror* (Zetterstedt, 1843)	LC	3	0	
*Cheilosiaurbana* (Meigen, 1822)	LC	7	0	
*Cheilosiauviformis* Becker, 1894	LC	1	0	
*Cheilosiavariabilis* (Panzer, 1798)	LC	3	0	
*Cheilosiavelutina* Loew, 1840	LC	1	0	
*Cheilosiavernalis* (Fallén, 1817)	LC	12	0	
*Cheilosiavicina* (Zetterstedt, 1849)	LC	2	0	
*Chrysogastersolstitialis* (Fallén, 1817)	LC	8	0	
*Chrysogastervirescens* Loew, 1854	NT	1	0	
*Chrysotoxum* Meigen, 1803		1	0	
*Chrysotoxumbicinctum* (Linnaeus, 1758)	LC	6	0	
*Chrysotoxumcautum* (Harris, 1776))	LC	12	8	
*Chrysotoxumcisalpinum* Rondani, 1845	VU	0	1	
*Chrysotoxumelegans* Loew, 1841	NT	6	0	
*Chrysotoxumfasciatum* (Muller, 1764)	LC	8	8	
*Chrysotoxumfasciolatum* (De Geer, 1776)	LC	1	0	
*Chrysotoxumfestivum* (Linnaeus, 1758)	LC	7	1	
*Chrysotoxumintermedium* Meigen, 1822	LC	4	0	
*Chrysotoxumlessonae* Giglio-Tos, 1890	[not evaluated]	1	0	
*Chrysotoxumoctomaculatum* Curtis, 1837	NT	3	0	
*Chrysotoxumvernale* Loew, 1841	LC	8	3	
*Copestylum* Macquart, 1846		1	0	
*Criorhinaranunculi* (Panzer, 1804)	LC	3	0	
*Dasysyrphusalbostriatus* (Fallén, 1817)	LC	1	0	
*Dasysyrphusneovenustus* Soszynski, Mielczarek & Tofilski, 2013	LC	1	0	
*Dasysyrphuspinastri* (De Geer, 1776)	LC	5	0	
*Dasysyrphustricinctus* (Fallén, 1817)	LC	2	0	
*Dasysyrphusvenustus* (Meigen, 1822)	LC	4	0	
*Dideaalneti* (Fallén, 1817)	LC	1	0	
*Dideafasciata* Macquart, 1834	LC	6	0	
*Epistrophediaphana* (Zetterstedt, 1843)	LC	1	0	
*Epistropheeligans* (Harris, 1780)	LC	10	0	
*Epistropheflava* Doczkal & Schmid, 1994	LC	1	0	
*Epistrophegrossulariae* (Meigen, 1822)	LC	5	0	
*Epistropheleiophthalma* (Schiner & Egger, 1853)	EN	3	0	
*Epistrophemelanostoma* (Zetterstedt, 1843)	LC	4	1	
*Epistrophenitidicollis* (Meigen, 1822)	LC	9	1	
*Epistropheobscuripes* (Strobl, 1910)	LC	1	0	
*Episyrphusbalteatus* (De Geer, 1776)	LC	15	32	
*Eristalinusaeneus* (Scopoli, 1763)	LC	14	4	
*Eristalinussepulchralis* (Linnaeus, 1758)	LC	5	0	
*Eristalisarbustorum* (Linnaeus, 1758)	LC	31	13	
*Eristalishorticola* (De Geer, 1776)	LC	9	0	
*Eristalisjugorum* Egger, 1858	LC	12	1	
*Eristalisnemorum* (Linnaeus, 1758)	LC	30	6	
*Eristalispertinax* (Scopoli, 1763)	LC	30	22	
*Eristalispicea* (Fallén, 1817)	LC	4	0	
*Eristalisrupium* Fabricius, 1805	LC	13	0	
*Eristalissimilis* (Fallén, 1817)	LC	10	0	
*Eristalistenax* (Linnaeus, 1758)	LC	50	35	
*Eumerus* Meigen, 1822		1	0	
*Eumerusfuneralis* Meigen, 1822	LC	1	0	
*Eumerusstrigatus* (Fallén, 1817)	LC	1	0	
*Eupeodes* Osten-Sacken, 1877		2	0	
*Eupeodescorollae* (Fabricius, 1794)	LC	14	1	
*Eupeodeslatifasciatus* (Macquart, 1829)	LC	4	0	
*Eupeodesluniger* (Meigen, 1822)	LC	5	0	
*Eupeodesluniger* (Zetterstedt, 1843)	LC	1	0	
*Eupeodesnitens* (Zetterstedt, 1843)	LC	1	0	
*Eupeodestirolensis* (Dusek & Laska, 1973)	NT	3	0	
*Eurimyialineata* (Fabricius, 1787)	LC	4	0	
*Ferdinandeaaurea* Rondani, 1844	LC	1	0	
*Ferdinandeacuprea* (Scopoli, 1763)	LC	1	0	
*Helophiluspendulus* (Linnaeus, 1758)	LC	12	28	
*Helophilustrivittatus* (Fabricius, 1805)	LC	5	3	
*Heringiaheringi* (Zetterstedt, 1843)	LC	1	0	
*Lapposyrphuslapponicus* (Zetterstedt, 1838)	LC	12	1	
*Leucozonaglaucia* (Linnaeus, 1758)	LC	8	0	
*Leucozonalaternaria* (Muller, 1776)	LC	3	0	
*Leucozonalucorum* (Linnaeus, 1758)	LC	8	1	
*Matsumyiaberberina* (Fabricius, 1805)	LC	1	0	
*Megasyrphuserraticus* (Linnaeus, 1758)	LC	3	0	
*Melangynaarctica* (Zetterstedt, 1838)	LC	1	0	
*Melangynacompositarum* (Verrall, 1873)	LC	8	0	
*Melangynalasiophthalma* (Zetterstedt, 1843)	LC	2	0	
*Melanogasterhirtella* (Loew, 1843)	LC	9	0	
*Melanogasternuda* (Macquart, 1829)	LC	2	0	
*Melanostomamellarium* (Meigen, 1822)	LC	2	0	
*Melanostomamellinum* (Linnaeus, 1758)	LC	16	10	
*Melanostomascalare* (Fabricius, 1794)	LC	19	13	
*Meligrammacincta* (Fallén, 1817)	LC	3	0	
*Meligrammacingulata* (Egger, 1860)	LC	2	0	
*Meligrammaeuchroma* (Kowarz, 1885)	LC	2	0	
*Meliscaevaauricollis* (Meigen, 1822)	LC	11	2	
*Meliscaevacinctella* (Zetterstedt, 1843)	LC	10	4	
*Merodon* Meigen, 1803		4	0	
*Merodonalbifrons* Meigen, 1822	LC	4	0	
*Merodonaureus* Fabricius, 1805	LC	2	0	
*Merodonavidus* (Rossi, 1790)	LC	1	0	
*Merodonclavipes* (Fabricius, 1781)	LC	3	0	
*Merodonequestris* (Fabricius, 1794)	LC	18	0	
*Merodonflavus* Sack, 1913	NT	9	0	
*Merodonmoenium* (Wiedemann, 1822)	LC	1	0	
*Merodonnigritarsis* Rondani, 1845	LC	1	0	
*Merodonruficornis* Meigen, 1822	LC	2	0	
*Merodonrufus* Meigen, 1838	LC	3	0	
*Microdonanalis* (Macquart, 1842)	NT	2	0	
*Microdonmutabilis* (Linnaeus, 1758)	VU	5	0	
*Milesiacrabroniformis* (Fabricius, 1775	LC	1	0	
*Myathropaflorea* (Linnaeus, 1758)	LC	16	13	
*Myoleptadubia* (Fabricius, 1805)	LC	2	0	
*Myoleptapotens* (Harris, 1776)	LC	1	0	
*Neoasciageniculata* (Meigen, 1822)	LC	4	0	
*Neoasciameticulosa* (Scopoli, 1763)	LC	8	0	
*Neoasciapodagrica* (Fabricius, 1775)	LC	2	0	
*Neoasciatenur* (Harris, 1780)	LC	5	0	
*Neocnemodonpubescens* (Delucchi & Pschorn-Walcher, 1955)	LC	1	0	
*Orthonevranobilis* (Fallén, 1817)	LC	4	0	
*Orthonevraonytes* (Séguy, 1961)	[not evaluated]	4	0	
*Paragus* Latreille, 1804		2	0	
*Paragusalbifrons* (Fallén, 1817)	EN	1	0	
*Paragusfinitimus* Goeldlin, 1971	EN	2	0	
*Paragushaemorrhous* Meigen, 1822	LC	1	1	
*Paragusstrigatus* Meigen, 1822	LC	1	0	
*Parasyrphusannulatus* (Zetterstedt, 1838)	LC	1	0	
*Parasyrphuslineola* (Zetterstedt, 1843)	LC	1	0	
*Parasyrphusmacularis* (Zetterstedt, 1843)	LC	6	1	
*Parasyrphusmalinellus* (Collin, 1952)	LC	4	0	
*Parasyrphuspunctulatus* (Verrall, 1873)	LC	14	0	
*Parasyrphusvittiger* (Zetterstedt, 1843)	LC	2	0	
*Pipiza* Fallén, 1810		4	0	
*Pipizaaustriaca* Meigen, 1822	LC	2	0	
*Pipizafasciata* Meigen, 1822	LC	2	0	
*Pipizafestiva* Meigen, 1822	LC	2	0	
*Pipizanocticula* (Linnaeus, 1758)	LC	1	0	
*Pipizaquadrimaculata* (Panzer, 1804)	LC	3	0	
*Pipizella* Rondani, 1856		6	0	
*Pipizelladivicoi* (Goeldlin, 1974)	LC	3	0	
*Pipizellaviduata* (Linnaeus, 1758)	LC	11	2	
*Pipizellazeneggenensis* (Goeldlin, 1974)	LC	2	0	
*Platycheirus* Le Peletier & Serville, 1828		3	0	
*Platycheirusalbimanus* (Fabricius, 1781)	LC	41	25	
*Platycheirusangustatus* (Zetterstedt, 1843)	LC	2	0	
*Platycheirusangustipes* Goeldlin, 1974	LC	1	0	
*Platycheirusclypeatus* (Meigen, 1822)	LC	3	0	
*Platycheirusdiscimanus* (Loew, 1871)	LC	2	0	
*Platycheirusimmaculatus* Ohara, 1980	LC	5	0	
*Platycheirusmanicatus* (Meigen, 1822)	LC	9	0	
*Platycheirusmelanopsis* Loew, 1856	LC	1	0	
*Platycheirusoccultus* Goeldlin, Maibach & Speight, 1990	LC	2	0	
*Platycheirusparmatus* Rondani, 1857	LC	7	0	
*Platycheirusscutatus* (Meigen, 1822)	LC	1	0	
*Platycheirusstictitus* (Meigen, 1822)	LC	1	0	
*Platycheirustarsalis* (Schummel, 1836)	LC	3	0	
*Pyrophaenagranditarsa* (Forster, 1771)	NT	1	0	
*Pyrophaenarosarum* (Fabricius, 1787)	LC	1	1	
*Rhingiacampestris* Meigen, 1822	LC	15	15	
*Rhingiarostrata* (Linnaeus, 1758)	LC	1	0	
*Scaevadignota* (Rondani, 1857)	LC	1	0	
*Scaevapyrastri* (Linnaeus, 1758)	LC	19	5	
*Scaevaselenetica* (Meigen, 1822)	LC	10	8	
*Sericomyiabombiformis* (Fallén, 1810)	LC	1	0	
*Sericomyialappona* (Linnaeus, 1758)	LC	8	0	
*Sericomyiasilentis* (Harris, 1776)	LC	7	1	
*Sericomyiasuperbiens* (Muller, 1776)	LC	3	0	
*Sphaerophoria* Le Peletier & Serville, 1828		8	0	
*Sphaerophoriainterrupta* (Fabricius, 1805)	LC	2	0	
*Sphaerophoriascripta* (Linnaeus, 1758)	LC	25	49	
*Spheginaclavata* (Scopoli, 1763)	LC	1	0	
*Spheginaclunipes* (Fallén, 1816)	LC	5	0	
*Spheginaelegans* Schummel, 1843	LC	1	0	
*Spheginalatifrons* Egger, 1865	LC	1	0	
*Spheginasibirica* Stackelberg, 1953	LC	5	0	
*Syrittapipiens* (Linnaeus, 1758)	LC	13	40	
*Syrphusnitidifrons* Becker, 1921	LC	2	0	
*Syrphusribesii* (Linnaeus, 1758)	LC	25	7	
*Syrphustorvus* Osten-Sacken, 1875	LC	22	2	
*Syrphusvitripennis* Meigen, 1822	LC	17	4	
*Temnostomabombylans* (Fabricius, 1805)	LC	5	1	
*Temnostomavespiforme* (Linnaeus, 1758)	LC	5	0	
*Trichopsomyiaflavitarsis* (Meigen, 1822)	LC	1	0	
*Triglyphusprimus* Loew, 1840	LC	1	0	
*Tropidiafasciata* Meigen, 1822	LC	2	0	
*Volucellabombylans* (Linnaeus, 1758)	LC	10	1	
*Volucellainanis* (Linnaeus, 1758)	LC	6	2	
*Volucellainflata* (Fabricius, 1794)	LC	5	0	
*Volucellapellucens* (Linnaeus, 1758)	LC	12	7	
*Volucellazonaria* (Poda, 1761)	LC	5	0	
*Xanthandruscomtus* (Harris, 1870)	LC	2	0	
*Xanthogrammacitrofasciatum* (De Geer, 1776)	LC	3	0	
*Xanthogrammadives* (Rondani, 1857)	LC	7	0	
*Xanthogrammapedissequum* (Harris, 1776)	LC	5	0	
*Xylotaflorum* (Fabricius, 1805)	LC	2	0	
*Xylotaignava* (Panzer, 1798)	LC	2	0	
*Xylotajakutorum* Bagatshanova, 1980	LC	2	0	
*Xylotasegnis* (Linnaeus, 1758)	LC	15	17	
*Xylotasylvarum* (Linnaeus, 1758)	LC	6	0	
*Xylotatarda* Meigen, 1822	LC	1	0	
**Total**		1302	423	

**Table 3.** New French Departmental data, according to Speight et al. (2024). The only *Chrysotoxumcisalpinum* record is from a card record, while all others are supported by at least one specimen.

Department	Species new for the Department
Ardèche	*Cheilosialaticornis*
Ardèche	*Leucozonalucorum*
Ardèche	*Pipizafasciata*
Aveyron	*Merodonalbifrons*
Aveyron	*Merodonnigritarsis*
Drôme	*Cheilosiaaerea*
Drôme	*Cheilosiaalbitarsis*
Drôme	*Cheilosiavernalis*
Drôme	*Chrysotoxumcisalpinum*
Drôme	*Eristalinussepulchralis*
Drôme	*Heringiaheringi*
Drôme	*Melangynalasiophthalma*
Drôme	*Merodonequestris*
Drôme	*Microdonmyrmicae*/*mutabilis*
Drôme	*Paragusalbifrons*
Drôme	*Platycheirusalbimanus*
Drôme	*Syrphusribesii*
Haute-Loire	*Cheilosiaaerea*
Haute-Loire	*Cheilosiaalbipila*
Haute-Loire	*Cheilosiacanicularis*
Haute-Loire	*Cheilosiahimantopus*
Haute-Loire	*Cheilosiaillustrata*
Haute-Loire	*Cheilosiaimpressa*
Haute-Loire	*Cheilosialatifrons*
Haute-Loire	*Cheilosiaorthotricha*
Haute-Loire	*Cheilosiavariabilis*
Haute-Loire	*Chrysogastersolstitialis*
Haute-Loire	*Chrysotoxumelegans*
Haute-Loire	*Chrysotoxumfasciolatum*
Haute-Loire	*Chrysotoxumintermedium*
Haute-Loire	*Chrysotoxumoctomaculatum*
Haute-Loire	*Chrysotoxumvernale*
Haute-Loire	*Dasysyrphusneovenustus*
Haute-Loire	*Dasysyrphuspinastri*
Haute-Loire	*Dasysyrphusvenustus*
Haute-Loire	*Dideafasciata*
Haute-Loire	*Epistropheflava*
Haute-Loire	*Epistrophegrossulariae*
Haute-Loire	*Epistrophenitidicollis*
Haute-Loire	*Eristalinussepulchralis*
Haute-Loire	*Eupeodesnitens*
Haute-Loire	*Lapposyrphuslapponicus*
Haute-Loire	*Leucozonalucorum*
Haute-Loire	*Melanostomascalare*
Haute-Loire	*Meligrammacincta*
Haute-Loire	*Meligrammaeuchroma*
Haute-Loire	*Paragusfinitimus*
Haute-Loire	*Paragushaemorrhous*
Haute-Loire	*Pipizafestiva*
Haute-Loire	*Pipizaquadrimaculata*
Haute-Loire	*Pipizellaviduata*
Haute-Loire	*Pipizellazeneggenensis*
Haute-Loire	*Platycheirusalbimanus*
Haute-Loire	*Platycheirustarsalis*
Haute-Loire	*Syrphustorvus*
Haute-Loire	*Volucellabombylans*
Haute-Loire	*Volucellazonaria*
Haute-Loire	*Xanthandruscomtus*
Haute-Loire	*Xanthogrammapedissequum*
Haute-Loire	*Xylotaignava*
Haute-Loire	*Xylotatarda*
Loire	*Brachyopapanzeri*
Loire	*Brachyopatestacea*
Isère	*Chalcosyrphusvalgus*
Isère	*Chrysotoxumlessonae*
Loire	*Cheilosiacanicularis*
Loire	*Cheilosialatifrons*
Loire	*Cheilosiapictipennis*
Loire	*Cheilosiasahlbergi*
Loire	*Cheilosiauviformis*
Loire	*Cheilosiavelutina*
Loire	*Chrysotoxumintermedium*
Loire	*Melangynaarctica*
Loire	*Melanostomamelliarium*
Loire	*Spheginaclavata*
Loire	*Spheginasibirica*
Rhône	*Epistrophediaphana*
Savoie	*Criorhinaberberina*
Savoie	*Xylotasegnis*
Vaucluse	*Cheilosiaantiqua*
Vaucluse	*Myoleptadubia*
Vaucluse	*Paragusstrigatus*
